# Supplementary material for: Excessive manganese content in children’s multivitamin supplements: Potential for neurodevelopmental harm and other adverse health outcomes
Source: PLoS One. 2026 Mar 18;21(3):e0343600. doi: 10.1371/journal.pone.0343600 (PMC12998863; doi:10.1371/journal.pone.0343600)
Supplement: S1 Table — (DOCX) [file pone.0343600.s001.docx]

Supplementary Table 1. Top 100 best-selling multivitamin and mineral supplements for children listed on Amazon.com (9 September 2025).

| **Product name** | **Mn on label** |
| --- | --- |
| Amazon Basics Kids' Multivitamin, 190 Gummies, Orange, Cherry & Strawberry (Previously Solimo) | no |
| Amazon Brand - Mama Bear Organic Kids Multivitamin, 60 Gummies, 1 Month Supply (Packaging May Vary), Berry, Cherry & Orange | no |
| Brain Booster+ 30mg Pure Saffron Extract Gummies & Multivitamin D3, B6, B12, Zinc, Supports Kids Brain Health, Focus, Memory, Attention, Mood Balance, Non-GMO Kids Supplement, 30x Servings | no |
| Carlyle Kids Multivitamin Gummies with Probiotics \| 60 Chewables \| Berry Flavor \| Vegetarian, Non-GMO, Gluten Free Children's Supplement \| by Lil' Sprouts \| Packaging May Vary | no |
| Centrum Kids Multivitamin Gummies, Tropical Punch Flavor Made With Natural Flavors, Stocking Stuffer, 150 Count, 150 Day Supply | no |
| CHILDLIFE ESSENTIALS Kids Multivitamin & Mineral - Kids Vitamins, Liquid Multivitamin for Kids, Toddler Multivitamins, Liquid Vitamins, Baby Multivitamin - Natural Orange & Mango, 8 Fl Oz (Pack of 1) | yes |
| Culturelle Kids Complete Chewable Multivitamin + Probiotic For Kids, Ages 3+, 50 Count, Digestive Health, Oral Health & Immune Support - With 11 Vitamins & Minerals, including Vitamin C, D3 & Zinc | no |
| Culturelle Kids Multivitamin + Probiotic For Kids (Ages 2+) - 60 Count, Peach-Orange & Mixed Berry Flavor - Digestive Health & Immune Support Gummies with Lutein to Support Eye Health | no |
| Culturelle Kids Probiotic + Complete Multivitamin Chewable For Kids, Ages 3+, 30 Count, Digestive Health, Oral Health & Immune Support - With 11 Vitamins & Minerals, including Vitamin C, D3 & Zinc | no |
| Dr. Berg Kids Chewable Multivitamins (NOT Sweetened w/Sugar) - Daily Multivitamin for Kids That Includes 20 Nutrients & Trace Mineral Complex - Mixed Berry Chewable Vitamins for Kid - Red Wafers | yes |
| Dr. Mercola Chewable Multivitamin for Kids, 30 Servings (60 Tablets), 0g Sugar Per Serving, Natural Orange Flavor, Dietary Supplement, Supports Overall Health, Non-GMO | yes |
| Emergen-C Kidz Vitamin C Immune Support Gummies Fun-Tastic Fruit 44CT includes Zinc, Manganese, B Vitamins and Vitamin D for Back to School Essentials | yes |
| Enfamil Poly-Vi-Sol Liquid Multivitamin Drops, Supports Growth & Immune Health*, 50mL Bottle​ | no |
| Enfamil Poly-Vi-Sol with Iron Liquid Multivitamin Drops, Supports Growth & Development*, 50mL Bottle​ | no |
| First Day Kids Multivitamin Gummies Age 4+, Strawberry & Orange - Kids Vitamins Multivitamin Gummy A C D3 K2 B1 B2 B6 B12 Folate, Immunity & Brain Function, Vegan - 30 Day Supply, Packaging May Vary | no |
| Flintstones Chewable Kids Vitamins, Complete Multivitamin for Kids and Toddlers with Iron, Calcium, Vitamin C, Vitamin D & More, 180ct | no |
| Flintstones Gummies Kids Vitamins with Immunity Support*, Kids and Toddler Multivitamin with Vitamin C, Vitamin D, B12, Zinc & more, Orange 150ct | no |
| Flintstones Sour Gummy Vitamins For Kids, Kids Multivitamin Gummies with Vitamins C, B6, B12, A & Vitamin D, Support Kids Growth and Development, 180 Count | no |
| Flintstones Vitamins Chewable Kids Multivitamin with + Extra Iron for Toddler & Kid with Vitamin C, D, Vitamin B12, 160 Count (Packaging Will Vary) | no |
| Flintstones Vitamins Complete Gummies, Kids Vitamins with Vitamin C for Toddlers and Children, Kids Multivitamin with Vitamin A, B6, B12, E & Zinc, 180 Count | no |
| Flintstones Vitamins for Picky Kids + Iron, Kids Multivitamin Gummies with Vitamin C, D, E, B6 and Iron, Gummy Vitamins, Grape Flavored, 60 Gummies | no |
| Focus Factor Kids Extra Strength Daily Chewable for Brain Health Support, 120 Count – Vitamins - Quality Formula – Gluten & Dairy Free Supplements for Children – No Artificial Sweetener | no |
| Garden of Life Kids Multivitamin Powder, Daily Vitamins and Minerals for Toddlers & Kids - Organic Toddler Multi Powder with 15 Vitamin C, D3, Zinc for Healthy Growth - Gluten Free - 30 Day Supply | yes |
| Garden of Life Organics Kids Gummy Vitamins - Fruit - Certified Organic, Non-GMO & Vegan Complete Children's Multi - B12, C & D3 - Gluten, Soy & Dairy Free, 120 Real Fruit Chew Gummies | yes |
| Gummy Vites Children's Chewable Gummy Bear Multivitamin Dietary Supplement, 300 Count | no |
| Halal Kids Multivitamin Gummies, 45-90 Days Supply, with All Essential Kids Vitamins. Toddler Vitamins with Vitamin C, D, Zinc, A, E, B6, B12, Biotin \| Halal Gummies for Kids | no |
| Halal Vitamins, Kids Vitamins, Kids Multivitamin Gummies Noor Vitamins: Vitamin C for Kids, D3, and Zinc for Immunity, B6 B12 for Energy. Non-GMO, Halal Gummies - 90 Count Gummy Vitamins for Kids | no |
| Height Growth Increase Gummies Vitamins, Grow Tall Supplement, Calcium, Magnesium, Zinc, Ashwagandha, Glucosamine Multivitamin for Teens, Kids, Adults | no |
| Height Growth Increase Vitamin Gummies, Calcium 100mg, Magnesium Glycinate 30mg, Zinc, Ashwagandha, Vitamin K2 D3 25mg Grow Tall Maximizer Supplement, Multivitamin for Kids, Teens Boy & Girl, Adults | no |
| HIYA Kids Multivitamin – 15+ Essential Vitamins & Minerals with Fruits & Veggies for Ages 2+, Supports Growth, Development & Immune, 0g Sugar – 20 Stick Packs for Toddlers & Kids | yes |
| Igennus Methylated Kids Multivitamin Gummy, MTHFR, No Artificial Sweeteners or Flavors, Sugar Free, Age 4+, 90 Natural Berry Flavor Gummies from Fruit Powder, Non-GMO, Halal, Clean Label | no |
| Iron Supplement Gummies for Kids - with Calcium, Zinc, Kids Multivitamin with Iron Gummies - Immune Support, Blood Builder & Energy Support for Iron Deficiency, Anemia, Vegan - 60 Gummies | no |
| JoySpring Burst B12 Drops - Vitamin B Complex for Kids (2 fl oz) B2, B6, B5 + Methylcobalamin B12 Children Supplement to Support Healthy Growth & Development, Vegan B12, Ages 2-16+ \| 60 Servings | no |
| JoySpring Methylfolate for Kids \| 5-MTHF Plus Methyl B12 with B6 (P5P) - MethylBee - MTHFR Supplement Kids B12 Vitamin \| Folinic Acid Alternative \| Sugar-Free, Berry Lemonade Drops -30 Servings | no |
| Kids Liquid Morning Multivitamin by MaryRuth's \| Kids Multivitamin \| Immune Support Supplement \| Multivitamin for Kids \| Vitamin C \| Vitamin D \| Essential Nutrients \| Vegan \| 15.22 fl oz | no |
| Kids Multivitamin Gummies - Sugar Free, Vegan, Non-GMO, Vitamin A, D, B6, B12 and C - No Artificial Sweeteners, Gluten-Free, Gelatin-Free - 60 Count (30 Day Supply) | no |
| Kids Multivitamin Gummies + Calcium, Probiotics, Vitamin D3, C, Methylated B12 & Folate, B6, A, K2, E, Zinc, Prebiotics for Children Ages 4+, Clean & Sugar Free, for Nutritional Support, 60 Cts | no |
| Kids Multivitamin Gummies with Iron, Sugar Free, Kids Daily Vitamins + Probiotics, Algae Omega 3 (EPA/DHA), A, C, D, Methyl B12, Folate, B6, Zinc, Biotin, for Healthy Growth, Immune Support, 60 Count | no |
| Kids Multivitamin Gummy with Iron + Probiotics for Immune Health & Digestion: Vitamins D, C, E, B, Zinc for Energy & Development, Vitamin Code, Non-GMO, Gluten-Free, 90 Orange Gummies, 30 Day Supply | no |
| Kids Multivitamin with Iron & Minerals Chewable Tablets - Mixed Fruit Flavor - Vegetarian, GMO-Free, Nut Free - Dietary Supplement - Digestive Support for Children - 120 Chewables", | no |
| L’il Critters Gummy Vites Daily Gummy Multivitamin for Kids, Vitamin C, D3 for Immune Support Cherry, Strawberry, Orange, Pineapple and Blueberry Flavors, 70 Count Gummies | no |
| L'il Critters Gummy Vites Daily Kids Multivitamins Assorted Fruit Flavors for Ages 2+ Toddlers & Kids, Gummy Vitamin with Vitamin C and D, 95-190 Day Supply, 190 Gummies | no |
| L’il Critters Gummy Vites No Sugar Added Daily Gummy Multivitamin for Ages 2+ Toddlers & Kids, Vitamin C, D3 for Immune, Strawberry and Orange Flavors, 100 Gummies | no |
| L’il Critters Paw Patrol Gummy Vites Daily Gummy Multivitamin for Kids, Vitamin C, D3 for Immune Support Cherry, Orange and Blueberry Flavors, 190 Gummies | no |
| L’il Critters Paw Patrol Gummy Vites Daily Gummy Multivitamin for Kids, Vitamin C, D3 for Immune Support Cherry, Orange and Blueberry Flavors, 60 Gummies | no |
| Lifeable Multivitamin & Multimineral with Iron Chewables for Kids – Vegetarian – Gluten Free Vegetarian – Great Tasting - Natural Flavored Pectin Chews with Vitamins A, B, C, D & E – 150 Count | no |
| Llama Naturals Kids Multivitamin Gummies, Vegan, Organic with Vitamin D, C & K, Toddler Real Fruit Gummy Vitamins, Chewable Supplement, Whole Food Multivitamin, No Added Sugar Cane, Strawberry, 90 Ct | no |
| MaryRuth Organics CoComelon Toddler Kids Multivitamin with Iron \| Immune Support \| Toddler Vitamins \| USDA Organic \| Sugar Free \| Multivitamin Liquid Drops for Kids Ages 1-3 \| Vegan \| 1 Fl Oz | no |
| MaryRuth Organics Kids Multivitamin Liposomal \| Vegan, Sugar-Free Vitamins for Kids \| Ages 4+ \| 2 Month Supply \| 15.22 Fl Oz | no |
| MaryRuth Organics Kids Vitamins \| Multi \| 1 Month Supply \| Multivitamin Gummies for Ages 4+ \| Multivitamin for Kids \| Vegan \| Only 2 Gummies a Day \| 60 Count | no |
| MaryRuth Organics Kids Vitamins \| Multi \| Sugar Free \| 2 Month Supply \| Kids Multivitamin Gummies for Ages 2+ \| Vegan \| Only 1 Gummy a Day \| 60 Count | no |
| MaryRuth Organics Kids Vitamins \| USDA Organic \| Multivitamin Gummies + Postbiotics for Ages 4+ \| Lactobacillus Rhamnosus \| Vegan \| Non-GMO \| 60 Count | no |
| MaryRuth Organics Liquid Morning Multivitamin for Women, Men & Kids \| Vitamin A C D E B6 B12 Biotin Zinc \| Beauty \| Vegan \| Non-GMO \| Gluten Free \| 32 Servings | no |
| MaryRuth Organics Sugar Free Vitamin Gummies for Kids Age 2 \| 2 Month Supply Multivitamin with Vitamin C, D3, Zinc \| 1 Gummy Per Day \| Toddler Vitamins \| Sugar Free \| Vegan \| Non GMO \| 60 Count | no |
| MaryRuth Organics Toddler Multivitamin Gummies + Postbiotics \| USDA Organic \| Vitamins for Kids Ages 2+ \| Immune Support \| Vitamin C \| Vitamin D3 \| Vitamin A \| Zinc \| Vegan \| Non-GMO \| 60 Servings | no |
| MaryRuth Organics Vitamin Gummy \| Kids and Toddlers Age 2+ \| USDA \| Daily Vitamin C \| D3 \| Zinc \| Mixed Berry and Cherry \| 2 Month Supply | no |
| Mega Liquid Multivitamins, Trace Minerals, Amino Acids, Turmeric for Adults, Men, Women, Teens, Kids, Non-GMO, No Soy, No Dairy | yes |
| MegaFood Kids One Daily Multivitamin - Convenient Mini Multivitamin for Kids - with Zinc, Vitamins C, B & D - Vegetarian, Gluten Free, Non GMO - 60 Tablets, 60 | yes |
| MegaFood Kids One Daily Multivitamin Soft Chews - Kids Vitamins with Vitamin B, Vitamin C, Vitamin D & Vitamin E - Age 4+, Vegetarian, Made Without 9 Food Allergens - Grape Flavor - 30 Chews | no |
| Mommy's Bliss Baby Multivitamin with Iron, Daily Essential Vitamins for Babies, Toddlers – Immune Support, Healthy Growth & Bone Development*, Age 2 Months+, 30 ml, Liquid | no |
| Nature Made Kids First Multivitamin with Omega-3, Kids Gummy Multivitamins with Vitamins and Minerals for Nutritional Support, 70 Gummies | no |
| NATURELO Chewable Vitamin for Kids – Multivitamin with Whole Food Organic Fruit Blend - 60 Tablets for Children | yes |
| Natures Plus Animal Parade Children's Chewable Multivitamin - 180 Animal-Shaped Tablets - Natural Assorted Flavors - Vegetarian, Gluten Free - 90 Servings | no |
| Nature's Way Alive! Children's Daily Chewable Multivitamin, Supports Bone, Eye, and Immune Health*, Orange & Berry Fruit Flavored, Gluten Free, 120 Chewable Tablets (Packaging May Vary) | yes |
| New Chapter Liquid Multivitamin + Mineral Supplement for Whole-Body Benefits, 100% Delicious Multivitamin for Adults, Teens & Kids 2+, Ready to Absorb Faster, Mixed Berry Flavored, 30 oz | yes |
| Nordic Naturals Nordic Berries, Cherry Berry - 120 Gummy Berries - Great-Tasting Multivitamin for Ages 2+ - Growth, Development, Optimal Wellness - Non-GMO, Vegetarian - 30 Servings | no |
| Nordic Naturals Nordic Berries, Citrus - 200 Gummy Berries - Great-Tasting Multivitamin for Ages 2+ - Growth, Development, Optimal Wellness - Non-GMO, Vegetarian - 50 Servings | no |
| NovaFerrum Yum \| Multivitamin with Iron for Infants, Toddlers & Kids \| Immune Support \| Ages 4 & Under \| Gluten Free Certified \| Sugar Free \| Raspberry Grape \| 120 Servings | no |
| NuBest Tall Kids - Toddlers Vitamins and Kids Vitamins for Age 2 to 9 - Support Bone Strength, Overall Health and Immunity - Animal Shapes - 90 Chewable Berry Tablets \| 6 Weeks Supply | no |
| Nutracelle NUTRAMIN Sugar-Free, Allergen-Free 100% Vegan Gummy Multivitamins for Kids - Great Tasting Natural Gummies Your Kids Will Love - 90 Count Bottle | no |
| NutraChamps Vitamin B Complex for Kids, B1, B2, B3, B6, B7, B9 & Methyl B12, Kids B Complex Liquid Drops Supplement, Energy, Focus, Metabolism, Natural Berry, 120 Servings, 4 Month Supply | no |
| OLLY Kids Multivitamin & Probiotic Gummy, Digestive Support, Vitamins A, D, C, E, B, Zinc, Chewable Supplement, Berry Flavor, 35 Day Supply - 70 Count | no |
| OLLY Kids Multivitamin & Probiotic Gummy, Digestive Support, Vitamins A, D, C, E, B, Zinc, Chewable Supplement, Berry Flavor, 60 day supply - 120 Count | no |
| OLLY Kids Multivitamin Gummy Worms, Overall Health and Immune Support, Vitamins and Minerals A, C, D, E, Bs and Zinc, Chewable Supplement, Sour Fruit Punch, 45 Day Supply (70 Count) | no |
| Omega 3 (EPA/DHA) Filled Gummies for Kids with Multivitamin, Calcium + D3 + K2 + Zinc, Plus Vitamin A B1 B2 B12 C, Selenium, lodine for Brain, Eye, Bone, Energy, No Fish Taste, Sugar Free | no |
| One A Day Kids Multivitamin Gummies, Kids Vitamins with Vitamin C, D, E & Zinc for Immune Support, Childrens Gummy Vitamins, Assorted Fruit Flavors, 60ct (Packaging May Vary) | no |
| One A Day Kids Multivitamin with Iron Gummy, Vitamin D, Vitamin C, E, Zinc for Immune Support, Gummy Vitamins, Kids Multivitamin Gummies with Iron, Cherry Flavor, 60 Count (Packaging May Vary) | no |
| Orgain Kids Sugar Free Multivitamin Gummies, Vegan & Plant Based, 50 Superfoods, 15 Vitamins and Minerals, Immune Support and 3g of Fiber, Mixed Berry, Ages 4+, 1 Month Supply (60 Gummies) | yes |
| Premium Liquid Multivitamin For Kids \| Sugar Free Kids Vitamins \| 100% DV of 14 Vitamins for Kids \| Multivitamin for Children Ages 4+ \| Great Tasting, Non-GMO, Max 98% Absorption Rate- 16 oz, 32 Serv | no |
| Pure Encapsulations Junior Nutrients - Kids & Teens Multivitamin - Without Iron - with Calcium, Vitamin D3 & Vitamin C - Non-GMO & Vegetarian - 120 Capsules | yes |
| Renzo's Picky Eater Kids Multivitamin with Iron, Dissolving Kids Vitamins with Vitamin D3 & K2 and More, 60 Sugar-Free Melty Tabs, Cherry Mo’ Cherry Flavored | no |
| Seeking Health Kids Multivitamin Chewable, Formulated for Children’s Health, with L-5-MTHF L-Methylfolate, B12, Riboflavin, Vitamin A, Vegetarian (60 Chewable Tablets)* | yes |
| SmartyPants Kids Multivitamin & Fiber Gummies: Prebiotic Fiber to Support Kids Digestion with Vitamin D3, C, Vitamin B12, B6, A, Zinc, Biotin, Folate, Three Fruit Flavors, 90 Count (30 Day Supply) | no |
| SmartyPants Kids Multivitamin & Immunity Gummies: Vitamins C, D, B6, B12, A, and Zinc for Immune Support, Biotin, Iodine, Gluten Free, Two Fruit Flavors, 60 Count (30 Day Supply) | no |
| SmartyPants Kids Multivitamin Gummies - Improved Formula: Omega 3 (DHA/EPA), Vitamins D3, C, B12, B6, Vitamin A, K & Zinc, Gluten Free, Three Fruit Flavors, 60 Count (30 Day Supply) | no |
| SmartyPants Kids Multivitamin Gummies, Sugar Free: Omega 3 (ALA), Vitamin D3, C, Vitamin B12, B6, A, K & Zinc for Immune Support, Biotin, Erythritol Free, Strawberry Banana, 44 Count (22 Day Supply) | no |
| SmartyPants Kids Multivitamin Gummies: Omega 3 Fish Oil (EPA/DHA), Vitamin D3, C, Vitamin B12, B6, A, K & Zinc for Immune Support, Biotin, Grape, Cherry & Berry Flavors, 120 Count (30 Day Supply) | no |
| SmartyPants Organic Toddler Multivitamin Gummies: Probiotics, Omega 3 (ALA), Vitamin D3, C, Vitamin B12, B6, A, K & Zinc, Beta Carotene, Gluten Free, Three Fruit Flavors, 60 Count (30 Day Supply) | no |
| SmartyPants Toddler Multivitamin Gummies - Improved Formula: Omega 3 (DHA/EPA), Vitamins D3, C, Vitamin B12, B6, A, K & Zinc, Biotin, Gluten Free, Three Fruit Flavors, 60 Count (30 Day Supply) | no |
| SOLARAY Childrens Vitamins & Minerals Complete Multivitamin for Kids Great Black Cherry Flavor (076280047974) (120 Chews, 60 Serv) | yes |
| Super Mini-Multi - Children's Multivitamin Swallowable Capsules with Methyl Folate, Methyl B12 and Coenzyme B Vitamins for Growth, Focus, Brain Health (120 Capsules) | yes |
| Tasteless Multivitamin Powder for Kids + Toddlers- No Sweeteners, Flavors, Colors or Preservatives. Add to Beverages or Food. Pure, Flavorless Children's Multivitamin Supplement. | no |
| Vitamin C Gummies 1000mg for Adults & Kids – Chewable Multivitamin with Zinc for Immune System & Collagen Support for Skin – Orange Flavor – Gluten Free, Non-GMO, Vegetarian – 60 Gummies | yes |
| Vitamin Friends - Vegan Multivitamin & Iron for Kids - Daily Nutritional Support Gummies w/Ferrous Fumarate B-Complex, Vitamin C, Zinc, Biotin - Body Function & Anemia - Strawberry, (60 Day Supply) | no |
| Youth Athletic Gummies – Kids Multivitamin Gummies with Essential Nutrients, Immune & Energy Support, Vegan & Gelatin-Free, Includes Vitamins A, C, B3, B12, Omega-3, Zinc – Ages 5-18 | no |
| YUM-V'S - Kids Multivitamin Chewable Chocolate Bears - Multivitamin for Kids Supplement - 16 Vitamins & Minerals - Vitamins Including D, Zinc, C, B Complex & More, 60 Count Pack of 1 | no |
| YumVs Kids Multivitamin with Iron & Minerals Chewable Tablets – Grape & Berry Flavor Vitamins for Toddlers & Children Age 2+ – Daily Multivitamin for Immune Support – Kosher, Non-GMO – 120 Chewables | no |
| Zahler Kids Multivitamin Chewable Vitamin Tablet - Complete One Daily Vitamins Supplement - Contains 20+ Minerals & Vitamins for Kids & Toddlers - Kosher Multivitamins Cherry Flavor (90) | yes |
| Zarbee’s Kids Elderberry Immune Support Supplement Gummies, Vitamins A C D3 E & Zinc, Elderberry Extract, Gluten & Gelatin Free, No Artificial Flavors, Natural Berry Flavor, Ages 2+, 42 Ct | no |
| Zarbee's Kid's Complete Daily Multivitamin + Probiotic Gummies with Vitamins A B C D E & zinc for Digestive Health Easy To Chew, Natural Fruit 70 Count | no |
