# Supplementary material for: Excessive manganese content in children’s multivitamin supplements: Potential for neurodevelopmental harm and other adverse health outcomes
Source: PLoS One. 2026 Mar 18;21(3):e0343600. doi: 10.1371/journal.pone.0343600 (PMC12998863; doi:10.1371/journal.pone.0343600)
Supplement: Attachment — Submitted filename: mn draft 14 AE COMMENTS.docx [file pone.0343600.s002.docx]

**Excessive manganese content in children’s multivitamin supplements: potential for neurodevelopmental harm and other adverse health outcomes**

Seth H. Frisbie^1*^, Erika J. Mitchell^2^, Amy Hoeltge^3^, Lindsey A. Pett^4^, Molly G. Hoeltge^5^

^1^ Department of Chemistry and Biochemistry, Norwich University, Northfield, VT, United States of America

^2^ Better Life Laboratories, Incorporated, East Calais, VT, United States of America

^3^ Department of General and Organic Chemistry, University of Vermont, Burlington, VT, United States of America

^4^ Department of Earth and Environmental Sciences, Norwich University, Northfield, VT, United States of America

^5^ Lake Champlain Waldorf School, Shelburne, Vermont, United States of America

* [sfrisbie@norwich.edu](mailto:sfrisbie@norwich.edu)

**Abstract**

*Background:* Manganese is an essential nutrient vital for many physiological processes, but it can cause adverse health effects at high exposures, particularly during neurodevelopment. Manganese (Mn) is included in many children’s multivitamin and mineral supplements, but it has not been documented whether the levels of Mn in such supplements are appropriate or safe.

*Methods:* We purchased 52 multivitamin and mineral supplements in various forms, including tablets, capsules, gummies, powders, and liquids. Only twenty-six (50%) of them listed supplemental Mn concentrations on their labels. After digesting the samples with nitric acid and hydrogen peroxide in a microwave digestor, we quantified their Mn concentrations using inductively coupled plasma-optical emission spectroscopy. We compared the measured Mn concentrations to the labeled concentrations and to the Institute of Medicine (IOM) Upper tolerable Levels (ULs).

*Results:* Of the 26 products with supplemental Mn concentrations on their labels, 23 (88.5%) contained more Mn than shown on the label, while 3 (11.5%) contained lower Mn concentrations. The mean percent difference between labeled and measured Mn content was 42.5%. One supplement (X%) exceeded the Institute of Medicine’s Mn ULs based on the labeled concentrations alone if consumed daily, 5 products (X%) would exceed ULs based on measured concentrations, and 9 (34.6% of the products with supplemental manganese listed on their labels) would exceed ULs based on measured concentrations if used as supplements to ordinary diets.

Of the 26 products without supplemental Mn listed on their labels, 13 (50.0%) contained detectable concentrations of Mn. None of the products without supplemental Mn listed on their labels would exceed ULs, even if consumed as supplements to ordinary diets.

*Conclusions:* Some children’s multivitamin supplements may lead to exceedances of Institute of Medicine’s ULs for Mn, especially when consumed as supplements to ordinary diets.

**Introduction**

Approximately one-third of children under the age of 19 years in the United States (US) use multivitamin supplements, according to National Health and Nutrition Examination Survey (NHANES) data from 2017 to 2018 [1]. Nearly 20% of the best-selling multivitamin supplements currently on the US market for children under 5 years indicate that they contain manganese on the label (Supplementary Table 1). Recent research has associated high manganese (Mn) exposures with various neurodevelopmental effects in children, while other studies have shown that low Mn exposures can also be potentially of concern [2]. Given that hundreds of thousands of children in the are exposed to Mn through daily multivitamin supplements, it is vital to ensure that the amounts of manganese contained in the children’s supplements are appropriate for healthy development.

In adults, excess Mn has long been recognized as a neurotoxin, causing manganism, which presents with balance issues, tremors, and mental and mood changes similar to Parkinson’s Disease [3]. Perhaps because Parkinson’s Disease is considered to be mainly a disease of older adults, it was not until recently that studies of excess exposures to Mn even included young adults or children as subjects [4].

However, once children’s neurodevelopment in the context of Mn exposures began to be investigated, researchers noted numerous neurodevelopmental problems associated with high Mn exposures, including behavioral problems, IQ deficits, attention deficits, and memory problems [2]. Significantly, U-shaped curves for Mn exposures were reported in some studies, with both low and high exposures associated with worse outcomes than moderate exposures [2]. Such U-shaped curves would be expected, given that Mn, while toxic at high doses, is also an essential nutrient, a critical component of many enzymes, and it also plays a role in bone formation and immune function.

Besides neurological problems, recent cross-sectional studies using data from large population surveys of health and nutrition, such as the NHANES in the US, have noted associations between high blood, serum, or urine Mn and anemia [5,6], decreased bone density [7-9], cardiovascular disease [10], kidney disease [11,12], liver disease [13-18], and sarcopenia [19].

While most of these studies examining associations between Mn biomarkers and health conditions included only adults, some studies examined adolescents and children. Studies focusing on data from children or adolescents noted associations between Mn biomarkers and anemia [20], decreased bone density [21], blood pressure and kidney parameters [22], hepatic steatosis [23], lung function [24], and dental caries [25].

Unfortunately, many of these cross-sectional studies did not investigate the possibility of negative health outcomes at both ends (high and low) of Mn exposure. Nevertheless, in the studies that did compare the extremes of exposures to more moderate exposures, U-shaped curves were commonly reported, including for kidney disease [11], anemia [6,20], cognitive performance [26], insulin resistance [27], diabetic retinopathy [28], kidney disease [11], liver disease [13], all-cause mortality [29], sarcopenia [30], and obesity [31]. For these studies identifying U-shaped curves describing the associations between Mn exposures and adverse health outcomes, inversion points were identified between 7.32 to 13.45 µg/L of Mn in blood [5,6,13,28-30,32].

Unfortunately, direct associations between dietary intakes of Mn and biomarkers (blood, serum, urinary concentrations) cannot be drawn from NHANES data because the NHANES food intake data do not include Mn content [33]. Other similar national surveys, such as the United Kingdom’s National Diet and Nutrition Survey (NDNS) and the Chinese Health and Nutrition Survey (CHNS), typically measure either Mn biomarkers or estimates of dietary intakes, but not both [34,35]. Nevertheless, in smaller studies that measured both dietary intakes of Mn and biomarkers of exposure, direct associations have been reported between high dietary intakes of Mn and low serum ferritin levels [36,37].

Manganese deficiency in humans eating normal diets is quite rare, and it is quite easy to obtain the required amounts of Mn in any diet that includes food intake [38], so Mn supplementation is likely unnecessary for most people. On the other hand, the risks of excessive Mn intakes are becoming better known as they are more thoroughly investigated. These risks may include neurodevelopmental harm for children, but also other health concerns such as anemia or reduced bone density [2,20].

Given the adverse health outcomes associated with high exposures to Mn, we believe it is important to quantify the amounts of Mn found in children’s multivitamin supplements. In this study, we measured the actual amount of Mn found in popular children’s multivitamin supplements and compared these amounts to the labeled values. We also calculated daily intakes of Mn due to supplements. We compared them to Adequate Intakes (AIs) and tolerable Upper intake Levels (ULs) published by the Institute of Medicine [39]. Our hypotheses were that the amounts of Mn contained in the children’s supplements would be accurately reflected on the labels and that daily Mn intakes from these supplements would help meet sufficiency requirements while not exceeding tolerable levels.

**Materials and Methods**

***Sample Selection***

A search using the term “children’s multivitamin with Mn” yielded 152 results at [www.amazon.com](http://www.amazon.com) on December 18, 2021. All 26 available products with “infants”, “babies”, “kids”, “toddlers”, or “children” and a Mn salt listed on their label were purchased. These 26 samples are assumed to be a reasonable representation of children’s multivitamins with supplemental Mn sold in the US.

This same search also yielded 48 products from 34 brands with “infants”, “babies”, “kids”, “toddlers”, or “children” and no Mn salt listed on their label. A random number generator was used to select 26 available products from 26 different brands. This second set of 26 samples was purchased and assumed to be a reasonable representation of children’s multivitamins without supplemental Mn sold in the US.

Thus, a total of 52 products were purchased from different brands in the US. Twenty-six of the 52 products listed a Mn salt listed on their labels. Of these 26 products, 3 were labeled “capsule”, 8 were labeled “chewable”, 5 were labeled “gummy”, 3 were labeled “liquid”, 3 were labeled “powder”, and 4 were labeled “tablet.” Twenty-six of these 52 products did not list a Mn salt listed on their labels. Of these 26 products, 2 were labeled “chewable”, 20 were labeled “gummy”, 3 were labeled “liquid”, and 1 was labeled “tablet.” Of the 52 products that we purchased, 50 listed 3 or more vitamins and minerals on the label, while 2 listed only a single vitamin or mineral on the label.

To assess the accuracy of our results, we also purchased Multielement Tablets Standard Reference Material® (SRM 3294) from the National Institute of Standards and Technology (NIST).

***Sample Preparation***

Solutions of all 52 samples and the Multielement Tablets Standard Reference Material® (SRM 3294) tablets were prepared for chemical digestion using laboratory-grade glassware. A microwave instrument (CEM Discover Explorer Hybrid 12®) with closed microwave vessels was employed for sample digestion. Approximately 0.200 to 0.230 grams (g) of each homogenized sample was weighed out in the reaction vessel and mixed with 10.00 milliliters (mL) of deionized water, 2.00 mL of concentrated nitric acid (HNO_3_; Fisher Chemical Lot 207552), and 0.50 mL of 30% hydrogen peroxide (H_2_O_2_; Fisher Chemical Lot 222582). The masses of these samples have 3 significant figures; therefore, most of the results in this paper are reported to the same precision. The vessels were reacted for approximately 5 minutes, then sealed and placed in the autosampler of the microwave instrument. The vessels were then heated to 120° Celsius (C) and maintained at that temperature for 30.0 minutes. The digestates were cooled to room temperature and made to 25.00 mL with deionized water. If needed, these digestates were subsequently diluted with additional deionized water to achieve a measured Mn concentration within the range used for calibration.

***Sample Analysis by Spectrophotometry***

The Mn concentrations were quantified by the 1-(2-pyridylazo)-2-naphthol (PAN) method [40]. The absorbance of the complexed Mn product was measured at 560 nanometers (nm) with a Hach 2010 spectrophotometer (Hach Company, Loveland, CO). Standard solutions at 0.00000, 0.17555, 0.35095, 0.52569, and 0.70173 mg/L of total Mn were used to calibrate the instrument. This work was done at Norwich University in Northfield, VT.

***Sample Analysis by Inductively Coupled Plasma-Optical Emission Spectroscopy***

Manganese concentrations were also quantified using inductively coupled plasma-optical emission spectroscopy with a SPECTRO ARCOS instrument (SPECTRO Analytical Instruments, Kleve, Germany). Each sample was introduced into the plasma as an aerosol via a nebulizer, where it was atomized and excited within an argon plasma operating at approximately 10,000 Kelvin (K). The SPECTRO ARCOS detected emitted light at element-specific wavelengths using an axial viewing configuration to optimize sensitivity and accuracy [41].

Standard solutions at 0.000, 5.006, 9.994, 14.997, and 19.983 mg/L of total Mn were prepared from an ISO 17034 and ISO 17025 certified stock solution (IV-28-125ML; Inorganic Ventures Lot T2-MEB724442) and were used by the instrument software for calibration. All standards and samples were analyzed in triplicate, and the mean concentration of each element was reported and used for statistical analysis. This work was also done at Norwich University in Northfield, VT.

***Quality Assurance and Quality Control***

Quality assurance and quality control measures included the analysis of the Multielement Tablets Standard Reference Material® (SRM 3294) from the National Institute of Standards and Technology in triplicate. In addition, after every analytical window of 13 samples, we also measured a reagent blank, a duplicate sample, and a known addition of standard to a sample (also called “sample matrix spikes” or “laboratory-fortified samples”). An additional reagent blank was analyzed immediately after calibration. Therefore, there were a total of 5 reagent blanks, 4 duplicate samples, and 4 known additions of standard to a sample during the analysis of all 52 samples. The analysis of the Standard Reference Material and the known additions of standard to samples were used to evaluate accuracy. The analysis of the duplicate samples was used to evaluate precision [42].

***Calculations and Statistical Analysis***

Differences between measured and labeled concentrations were calculated by subtracting the labeled values from the measured concentrations. Percent differences between measured and labeled concentrations were calculated by dividing the absolute value of the differences by the average of the differences and multiplying by 100. Thus, percent differences are always positive. Percent differences were only calculated for products with labeled concentrations of Mn.

Statistical analyses were performed in R version 4.5.1, “Great Square Root.” Normality of data was checked with the Shapiro-Wilk test prior to performing any hypothesis testing. Non-parametric methods (Wilcoxon, Spearmann rank correlation) were used for hypothesis testing for data with non-normal distributions. A significance level of *p*<.05 was assumed for all statistical tests.

**Results and Discussion**

***Accuracy and Precision of the Determination of Mn Results by Spectrophotometry***

The percent recoveries of known additions of standard to sample 2 and sample 2 duplicate are 98.6% and 101%, respectively (Table 1). These percentages suggest that all the Mn we added to sample 2 was recovered. It also suggests that our measured concentration of Mn by spectrophotometry, at least for sample 2, is highly accurate.

Table 1. The percent recoveries of known additions of standard to samples measured by spectrophotometry.

| **Sample** | **Percent**  **Recovery (%)** |
| --- | --- |
| 2 | 98.6% |
| 2 Duplicate | 101% |
| 20 | −4.74% |
| 20 Duplicate | −3.43% |
| 28 | 4.00% |
| 28 Duplicate | 1.52% |
| 52 | −3.50% |
| 52 Duplicate | 20.3% |

In contrast, percent recoveries of known additions of standard to sample 20, sample 20 duplicate, sample 28, sample 28 duplicate, sample 52, and sample 52 duplicate are −4.74%, −3.43%, 4.00%, 1.52%, −3.50%, and 20.3%, respectively (Table 1). These percentages suggest that for all practical purposes, none of the Mn we added to samples 20, 28, and 52 was recovered. It also suggests that our measured concentrations of Mn by spectrophotometry, at least for samples 20, 28, and 52, are inaccurate.

In conclusion, the percent recoveries of known additions of standard to samples measured by spectrophotometry are unsatisfactory and cannot be used to measure the actual concentrations of Mn in these 52 children’s vitamins. Accordingly, we decided to measure the actual concentrations of Mn in these 52 children’s vitamins by inductively coupled plasma-optical emission spectroscopy. In spectrophotometry, the aqueous Mn ion (Mn^2+^_(aq)_) reacts with a colorizing agent under relatively mild conditions at room temperature. In contrast, inductively coupled plasma-optical emission spectroscopy involves converting the sample into individual atoms using an argon plasma that reaches temperatures around 10,000 Kelvin. This separation of atoms at high-energy conditions significantly reduces the likelihood of Mn binding to any other atom [41].

***A Hypothesis About the Bioavailability of Mn in Some of the Children’s Supplement Products in our Study***

These unsatisfactory percent recoveries of known additions of standard to samples by spectrophotometry motivated us to repeat these analyses (Table 1). The results of these repeated analyses still had poor recoveries of added Mn. The raw data from these analyses are in the Supporting Information section (Supplementary Table 2). Essentially, none of the Mn that we added to samples 20, 28, and 52 was recovered. This suggests that the digestates of these 3 samples remove the added Mn from solution and tightly bind it to the sample matrix.

Given this loss of added Mn during quality assurance testing, it is possible that the Mn added by the manufacturer to these products might also be tightly bound to other components in the tablets or powders. If this is the case, the Mn in these products may not be bioavailable. In fact, some products might remove Mn and possibly other minerals from a child’s body. This possible lack of bioavailability and the possible removal of minerals from a child’s body could result in a net loss of nutrients rather than a gain.

It is important to note that the sample digestate differs from human digestate. The samples were digested at 120°C for 30.0 minutes using nitric acid and hydrogen peroxide, which are relatively vigorous conditions. In contrast, human digestion occurs at approximately 37°C under milder conditions [43]. As a result, the chemical composition of these 2 digestates varies. However, it is still possible that some of these children’s products in the human digestive system may not release Mn for metabolism and may instead remove Mn and other minerals from the body.

***The Detection Limit, Accuracy, and Precision of the Determination of Mn by Inductively Coupled Plasma-Optical Emission Spectroscopy***

The detection limit is the smallest concentration that can be reported with reasonable certainty for a given analytical procedure [44]. There are many ways to calculate a detection limit [45]. For this study, we defined our detection limit as the upper limit of a 1-tailed 99% confidence interval from the 5 separately prepared 0.00 mg/L Mn standards that were analyzed as samples. This approach is called the detection limit based on control charts [46]. This detection limit was 0.001 mg/L for digestates as analyzed by ICP-OES, which equals 0.0001 mg/g for a sample of supplement product when 0.200 g of this sample is digested and diluted to 25.00 mL. These detection limits are rounded to one significant figure.

The measured concentrations of Mn in 3 analyses of the Multielement Tablets Standard Reference Material (SRM 3294) were 1.36 milligrams per gram of tablet (mg/g), 1.43 mg/g, and 1.44 mg/g by inductively coupled plasma-optical emission spectroscopy. All 3 concentrations fall within the acceptable range of 1.33 mg/g to 1.55 mg/g established by the National Institute of Standards and Technology [47]. Therefore, the measured Mn concentrations for all 52 samples analyzed by inductively coupled plasma-optical emission spectroscopy are considered accurate according to this first definition of accuracy [42].

The percent recoveries of known additions of standard to samples were measured at 92.7%, 99.0%, 102%, and 112% by inductively coupled plasma-optical emission spectroscopy. The 95% confidence interval for these recoveries ranges from 88.7% to 114%. Since this range includes 100%, the measured Mn concentrations for all 52 samples analyzed by inductively coupled plasma-optical emission spectroscopy are considered accurate according to this second definition of accuracy [42].

The precision of samples, based on the analysis of 4 samples in duplicate, is 0.007 mg/g. This precision is excellent, given that it does not vary until the thousandths of a mg/g. In contrast, the Multielement Tablets Standard Reference Material (SRM 3294) varies at the hundredths of a mg/g [42,47].

In conclusion, the percent recoveries of the Multielement Tablets Standard Reference Material (SRM 3294) by inductively coupled plasma-optical emission spectroscopy are highly accurate. The known additions of standard to samples measured by this method are highly accurate. The precision of samples by this method is highly precise. Therefore, inductively coupled plasma-optical emission spectroscopy was used to measure and report the actual concentrations of Mn in the 52 children’s vitamins in this study.

***The Mn Concentrations Reported on Labels Compared to the Concentrations Measured in Samples***

Labeled and measured concentrations of Mn for the 52 children’s supplement products are shown in Table 2.

Table 2. Labeled and measured concentrations of manganese in the 52 children’s supplement products in milligrams of manganese (Mn) per gram of product (mg/g), the percent differences between labeled and measured manganese content, and the costs per day for a 5-year-old. Samples with measured concentrations below the nominal 0.0001 mg/g detection limit are labeled as ND (Non-Detect). All concentrations are reported to 3 significant figures or to the nearest 0.0001 of a mg/g.

| **Sample** | **Labeled Mn (mg/g)** | **Measured Mn (mg/g)** | **Difference between Labeled and measured Mn (mg/g)** | **% Difference between Labeled and Measured Mn** | **Cost per Day for 5-Year-Old** |
| --- | --- | --- | --- | --- | --- |
| 1 | 1.79 | 2.52 | 0.729 | 33.9% | $0.34 |
| 2 | 1.15 | 0.737 | −0.410 | 43.5% | $0.41 |
| 3 | 0.708 | 0.832 | 0.124 | 16.2% | $0.41 |
| 4 | 0.740 | 0.831 | 0.0910 | 11.6% | $0.17 |
| 5 | 0.696 | 0.894 | 0.198 | 24.8% | $0.20 |
| 6 | 0.804 | 0.904 | 0.100 | 11.7% | $0.77 |
| 7 | 0.565 | 0.656 | 0.0914 | 15.0% | $0.65 |
| 8 | 0.611 | 0.561 | −0.0499 | 8.51% | $0.50 |
| 9 | 0.167 | 0.192 | 0.0256 | 14.3% | $0.32 |
| 10 | 0.133 | 0.299 | 0.166 | 76.8% | $0.43 |
| 11 | 0.158 | 0.217 | 0.0593 | 31.6% | $0.14 |
| 12 | 0.138 | 0.172 | 0.0346 | 22.3% | $0.99 |
| 13 | 0.115 | 0.245 | 0.130 | 72.4% | $0.31 |
| 14 | 0.0303 | 0.0473 | 0.0170 | 43.8% | $0.42 |
| 15 | 0.0041 | 0.0064 | 0.0023 | 43.7% | $0.43 |
| 16 | 0.0041 | 0.0067 | 0.0026 | 49.1% | $0.38 |
| 17 | 0.136 | 0.158 | 0.0213 | 14.5% | $1.69 |
| 18 | 0.0029 | 0.0071 | 0.0042 | 84.6% | $0.43 |
| 19 | 0.0001 | 0.0371 | 0.0370 | 199% | $0.70 |
| 20 | 0.144 | 0.376 | 0.230 | 88.8% | $0.96 |
| 21 | 0.0532 | 0.0939 | 0.0407 | 55.4% | $0.50 |
| 22 | 0.125 | 0.0957 | −0.0293 | 26.5% | $0.89 |
| 23 | 0.753 | 0.856 | 0.103 | 12.8% | $0.28 |
| 24 | 0.378 | 0.724 | 0.346 | 62.8% | $0.37 |
| 25 | 0.556 | 0.692 | 0.136 | 21.7% | $0.14 |
| 26 | 0.726 | 0.879 | 0.153 | 19.1% | $0.42 |
| 27 |  | 0.0280 |  |  | $0.07 |
| 28 |  | ND |  |  | $0.56 |
| 29 |  | ND |  |  | $0.67 |
| 30 |  | 0.0002 |  |  | $0.31 |
| 31 |  | 0.0004 |  |  | $0.73 |
| 32 |  | ND |  |  | $0.74 |
| 33 |  | 0.108 |  |  | $0.32 |
| 34 |  | ND |  |  | $0.14 |
| 35 |  | 0.005 |  |  | $0.87 |
| 36 |  | ND |  |  | $0.53 |
| 37 |  | 0.0002 |  |  | $0.13 |
| 38 |  | 0.0002 |  |  | $0.33 |
| 39 |  | ND |  |  | $0.44 |
| 40 |  | ND |  |  | $0.30 |
| 41 |  | 0.0063 |  |  | $0.68 |
| 42 |  | ND |  |  | $0.13 |
| 43 |  | 0.0002 |  |  | $0.13 |
| 44 |  | 0.0002 |  |  | $0.27 |
| 45 |  | ND |  |  | $0.50 |
| 46 |  | ND |  |  | $0.47 |
| 47 |  | 0.0024 |  |  | $0.49 |
| 48 |  | 0.0005 |  |  | $0.50 |
| 49 |  | ND |  |  | $0.28 |
| 50 |  | ND |  |  | $1.70 |
| 51 |  | 0.0007 |  |  | $0.35 |
| 52 |  | ND |  |  | $0.87 |
| Mean for Mn labeled products | 0.418 | 0.501 | 0.0905 | 42.5% | $0.51 |
| Mean for products without labeled Mn |  | 0.0117 |  |  | $0.48 |
| Mean for Mn labeled products, omitting sample #19 |  |  |  | 36.2% |  |
| Minimum for Mn labeled products | 0.0001 | 0.0064 | −0.410 | 8.51% | $0.14 |
| Maximum for Mn labeled Products | 1.79 | 2.52 | 0.729 | 199% | $1.69 |
| Minimum for products without labeled Mn |  | ND |  |  | $0.07 |
| Maximum for products without labeled Mn |  | 0.108 |  |  | $1.70 |

As shown in Table 2, 23 of the 26 (88.5%) products with a Mn salt listed on their labels contained more Mn than was shown on the label, while 3 (11.5%) products contained less. One product (sample 19) appeared to have a typographical error in the label; the Mn content was claimed to be “1 mcg” (interpreted as 1 microgram, µg) per serving when most likely “1 mg” (1 milligram, mg) was intended. This likely typographical error resulted in a 199 percent difference between the measured and labeled Mn concentrations for this sample (Table 2). The mean percent difference between the measured and labeled concentrations for the remaining 25 products with a Mn salt listed on their labels was 36.2%. The mean percent differences between labeled and measured Mn (42.5% for all samples or 36.2% excluding the sample with the presumed typographical error) were considerably higher than the 24% mean percent difference between labeled and measured Mn content reported by the US Department of Agriculture survey of children’s multivitamin products published in 2017 [48].

Notably, 13 of the 26 (50.0%) products that did not have Mn on the label contained measurable concentrations of Mn. The mean measured concentration of Mn in the products with no Mn salt listed on their labels was 0.0059 mg/g, while the mean measured concentration in the products with a Mn salt listed on their labels was 0.501 mg/g; this difference was statistically significant (W(52) = 10, *p* < .001).

The cost of a one-day supply of product for a 5-year-old ranged from $0.07 to $1.70. There was no difference in average cost between products with a Mn salt listed on their labels ($0.51) and those with no Mn salt listed on their labels ($0.48) (W(52) = 319, *p*=.735). The cost of a one-day supply was also not correlated with the magnitude of difference between the labeled and measured Mn content (*r_s_*(50) = -0.13, *p* = .368).

***Regulations Applicable to Mn in Children’s Supplements in the US***

Dietary supplements, including children’s multivitamins, are regulated in the US by the Dietary Supplement Health and Education (DSHE) Act of 1994 [49]. This act states that the government should not “impose unreasonable regulatory barriers limiting or slowing the flow of safe products to consumers.” To avoid imposing regulatory barriers, the Dietary Supplement Health and Education Act classifies dietary supplements as foods; thus, they are not subject to the special regulations that apply to drugs and medications.

Regarding safety, the government reserves the right to deem supplements adulterated that present “significant or unreasonable risks of illness or injury” under “conditions of use recommended or suggested in labelling” [49]. However, the burden of proof of adulteration, according to such a definition, is placed specifically with the government. Thus, there are no specific regulations regarding Mn content in children’s supplements in the US. Manufacturers are forbidden from selling unsafe products. However, if a product should turn out to be unsafe, the government must provide specific evidence of the risks and harms before it can deem the product adulterated and remove it from the market.

***The Generally Recognized as Safe List and the Labeled Supplemental Mn Ingredients***

The US Food and Drug Administration (U.S. FDA) maintain a list of food additives that are Generally Recognized as Safe (GRAS) [50]. Food additives that do not appear on this list require pre-approval by the FDA before use. For Mn, Mn citrate, Mn dichloride, Mn gluconate, Mn glycerophosphate, manganese(II) hypophosphite, and manganese(II) sulfate are on the GRAS list [50]. Five samples contained forms of Mn not on the Food and Drug Administration’s GRAS list, including 3 that contained Mn amino acid chelate (samples 7, 11, 13) and 2 that contained Mn bisglycinate (samples 14, 26).

***Governmental Recommendations Applicable to the Mn Content in Children’s Supplements***

The National Institutes of Health (NIH) in the US relies on the recommendations of the Food and Nutrition Board (FNB) of the National Academies of Sciences Engineering, and Medicine (NASEM), a congressionally chartered non-profit institution, for its Daily Reference Intakes (DRIs). In the case of Mn, these recommendations include Adequate Intakes (AIs) and tolerable Upper intake Levels (ULs) [51,52]. The National Academies of Sciences, Engineering, and Medicine’s DRIs were originally developed by the Institute of Medicine (IOM), so they are often referred to in the literature as the Institute of Medicine’s DRIs [39]. The Institute of Medicine’s DRIs for Mn for infants and children 8 years old and younger are shown in Table 3 [39].

Table 3. The Institute of Medicine’s Dietary Reference Intakes (DRIs) for manganese for infants and children 8 years old and younger [51,52].

| **Age** | **Adequate Intakes (AIs)** | **Tolerable Upper Intake Levels (ULs)** |
| --- | --- | --- |
| 0-6 months | 0.003 mg/day | No Data |
| 7-12 months | 0.6 mg/day | No Data |
| 1-3 years | 1.2 mg/day | 2 mg/day |
| 4-8 years | 1.5 mg/day | 3 mg/day |

These AIs and ULs were drawn from a review of nutritional studies published in 2001[39]; thus, they do not reflect any of the vast research on Mn sufficiency and toxicity published over the last 25 years. The Institute of Medicine did not find data specific to nutritional adequacy for Mn, so they based the AIs on the median recorded intakes of Mn in food [39]. That is, the median intakes were redefined as the adequate intakes, so individuals not consuming the median amounts were classified as undernourished with regard to Mn. This decision would logically tend to increase the median Mn intake across populations actively seeking “sufficiency.”

For the ULs in children over 1 year old, the Institute of Medicine scaled down its adult UL of 11 mg/day by weight to apply to children. For infants less than 1 year old, the scaling approach was not used due to “concern about the infant’s ability to handle excess amounts.” The Institute of Medicine goes on to remark, “To prevent high levels of Mn intake, the only source of intake for infants should be from food or formula” [39]. It must be kept in mind that the adult UL of 11 mg/day was also derived as the maximum intake from a dietary study that did not measure any health outcomes; this maximum intake of 11 mg/day was consumed by a total of 2 adult women during a 3-day dietary survey, while the remaining 98 women in the study consumed lower amounts of Mn [39,53].

Manganese metabolism is quite complex, with body levels of Mn controlled by homeostatic mechanisms [54]. Relatively high intakes of Mn from food or beverages such as tea have not been found to be directly correlated with blood or serum Mn levels [55]. In contrast, Mn intakes through drinking water or Mn supplements have been correlated with elevated blood, serum, or hair Mn levels, suggesting that the Mn from supplements may be more readily absorbed than regular dietary Mn consumed in a complex matrix of other nutrients [56-59].

In sum, the AIs used in the US are based not on actual sufficiency, but rather on middle measures of dietary intakes (medians), so they should more accurately be termed “average intakes” rather than “adequate intakes.” Intakes near these levels that are below the stated AIs may not actually be inadequate, but might more accurately be termed “below average intakes.” More data are needed to determine the nutritional adequacy of Mn, particularly data identifying blood Mn levels that protect against adverse health outcomes and dietary patterns that might lead to these levels.

In contrast, the ULs are based on maximum adult dietary intakes from a single study lacking associated health data [39,53]. Extremely high blood Mn levels have been associated with a variety of adverse health outcomes. If there should turn out to be any predictive relationships between dietary Mn and blood Mn, then the extreme dietary Mn intakes from the single study used to define the ULs may not be entirely without risk [53]. Certainly, if children, who are actively in the process of neurological and physical development, were to proportionately exceed these maximum recorded adult dietary intakes through consumption of supplemental Mn, this would potentially be of concern.

**Daily Exposure Estimates for Mn**

***Daily Exposure Estimates Based on Labeled Values***

Daily exposure estimates based on labeled Mn content for each age for which the samples are labeled for use are shown in Table 4.

Table 4. Daily exposure estimates based on the labeled manganese content at different ages, ranging from 6 months to 5 years. Only samples with labeled manganese content are included. Separate estimates are provided for each age covered by the indications on the label. Labeled values which exceed the Institute of Medicine’s tolerable Upper intake Levels (ULs) for corresponding age classes are highlighted in **bold**. Labeled values equal to the Institute of Medicine’s ULs are highlighted with ***bold italics***.

| **Sample** | **Daily Intakes of Manganese Based on Labeled Values (mg/day)** | | | | | |
| --- | --- | --- | --- | --- | --- | --- |
|  |  |  |  |  |  |  |
|  | **6 Months** | **1 Year** | **2 Years** | **3 Years** | **4 Years** | **5 Years** |
|  |  |  |  |  |  |  |
| ***1*** |  |  | ***2*** | ***2*** | 2 | 2 |
| 2 |  |  |  |  | 1 | 1 |
| 3 |  | 0.375 | 0.375 | 0.375 | 0.75 | 0.75 |
| ***4*** |  |  | ***2*** | ***2*** | 2 | 2 |
| 5 |  |  | 1.15 | 1.15 | 2.3 | 2.3 |
| 6 |  |  |  |  | 2 | 2 |
| 7 |  |  | 1 | 1 | 2 | 2 |
| 8 |  |  |  |  | 1 | 1 |
| 9 |  |  |  | 0.5 | 0.5 | 0.5 |
| 10 |  | 0.3 | 0.3 | 0.3 | 0.3 | 0.3 |
| 11 |  |  |  |  | 0.25 | 0.25 |
| 12 |  |  |  |  | 0.9 | 0.9 |
| 13 |  |  |  |  | 0.6 | 0.6 |
| 14 |  |  |  |  | 0.1 | 0.1 |
| 15 |  |  | 0.01 | 0.01 | 0.02 | 0.02 |
| 16 |  |  |  | 0.01 | 0.02 | 0.02 |
| **17** | **0.8** | 1.5 | 1.5 | 1.5 | 2.3 | 2.3 |
| 18 |  | 0.05 | 0.05 | 0.05 | 0.05 | 0.05 |
| 19 |  |  | 0.001 | 0.001 | 0.001 | 0.001 |
| 20 |  |  |  |  | 1.5 | 1.5 |
| 21 |  |  |  |  | 0.5 | 0.5 |
| 22 |  |  | 0.25 | 0.25 | 0.25 | 0.25 |
| ***23*** |  |  | ***2*** | ***2*** | 2 | 2 |
| 24 |  |  |  |  | 1 | 1 |
| 25 |  |  | 0.5 | 0.5 | 1 | 1 |
| 26 |  |  |  |  |  | 0.4 |

Of the 26 products with a Mn salt listed on their labels, 1 out of 26 (3.85%) products exceeded an Institute of Medicine’s UL. This product was labeled for use by infants 6 months and older and provides 0.8 mg of Mn per day for infants 6 to12 months old. For infants below 1 year old, the Institute of Medicine notes, “To prevent high levels of Mn intake, the only source of intake for infants should be from food or formula” [39]. Thus, any supplement containing detectable amounts of Mn exceeds the Institute of Medicine recommendation for this age.

An additional 3 out of 26 (11.5%) products have labeled Mn content that equals the Institute of Medicine’s UL for specified ages (Tables 3 and 4). While these labeled values do not exceed the Institute of Medicine’s ULs, they leave no room for any further Mn exposures through either food or water. Consumption of any food or water that contains Mn in conjunction with a daily dose of one of these supplements will lead to exceedances of the Institute of Medicine’s ULs, based on the labeled content of the supplements.

***Daily Exposure Estimates Based on Measured Values***

Daily exposure estimates based on measured Mn content for each age for which the samples are labeled for use are shown in Table 5.

Table 5. Daily exposure estimates in mg/day based on measured Mn content at different ages from 6 months to 5 years. Separate estimates are provided for each age covered by indications on the label. Products that exceed the Institute of Medicine’s ULs for corresponding age classes based on measured Mn content are highlighted in **bold**. Samples with measured Mn contents below the detection limit are omitted.

| **Sample** | **Daily Intakes of Manganese (Mn) Based on Measured Values** | | | | | |
| --- | --- | --- | --- | --- | --- | --- |
|  | **Daily Intake** | **Daily Intake** | **Daily Intake** | **Daily Intake** | **Daily Intake** | **Daily Intake** |
|  | **6 Months** | **1 Year** | **2 Years** | **3 Years** | **4 Years** | **5 Years** |
|  | **(mg/day)** | **(mg/day)** | **(mg/day)** | **(mg/day)** | **(mg/day)** | **(mg/day)** |
| **1** |  |  | **2.82** | **2.82** | 2.82 | 2.82 |
| 2 |  |  |  |  | 0.643 | 0.643 |
| 3 |  | 0.441 | 0.441 | 0.441 | 0.882 | 0.882 |
| **4** |  |  | **2.25** | **2.25** | 2.25 | 2.25 |
| 5 |  |  | 1.48 | 1.48 | 2.95 | 2.95 |
| 6 |  |  |  |  | 2.25 | 2.25 |
| 7 |  |  | 1.16 | 1.16 | 2.32 | 2.32 |
| 8 |  |  |  |  | 0.918 | 0.918 |
| 9 |  |  |  | 0.577 | 0.577 | 0.577 |
| 10 |  | 0.674 | 0.674 | 0.674 | 0.674 | 0.674 |
| 11 |  |  |  |  | 0.344 | 0.344 |
| 12 |  |  |  |  | 1.13 | 1.13 |
| 13 |  |  |  |  | 1.28 | 1.28 |
| 14 |  |  |  |  | 0.156 | 0.156 |
| 15 |  |  | 0.0156 | 0.0156 | 0.0312 | 0.0312 |
| 16 |  |  |  | 0.0165 | 0.0330 | 0.0330 |
| **17** | **0.925** | 1.85 | 1.85 | 1.85 | 2.78 | 2.78 |
| 18 |  | 0.123 | 0.123 | 0.123 | 0.123 | 0.123 |
| 19 |  |  | 0.610 | 0.610 | 0.610 | 0.610 |
| **20** |  |  |  |  | **3.10** | **3.10** |
| 21 |  |  |  |  | 0.950 | 0.950 |
| 22 |  |  | 0.205 | 0.205 | 0.205 | 0.205 |
| **23** |  |  | **2.27** | **2.27** | 2.27 | 2.27 |
| 24 |  |  |  |  | 1.92 | 1.92 |
| 25 |  |  | 0.622 | 0.622 | 1.24 | 1.24 |
| 26 |  |  |  |  |  | 0.484 |
| 27 |  |  | 0.0191 | 0.0191 | 0.0382 | 0.0382 |
| 30 |  | 0.0012 | 0.0012 | 0.0012 | 0.0012 | 0.0012 |
| 31 |  |  | 0.0009 | 0.0009 | 0.0017 | 0.0017 |
| 33 |  |  | 0.324 | 0.324 | 0.648 | 0.648 |
| 35 |  |  | 0.0117 | 0.0117 | 0.0235 | 0.0235 |
| 37 |  |  | 0.0005 | 0.0005 | 0.0010 | 0.0010 |
| 38 |  |  | 0.0013 | 0.0013 | 0.0013 | 0.0013 |
| 41 |  |  |  |  | 0.0250 | 0.0250 |
| 43 |  |  | 0.0004 | 0.0004 | 0.0009 | 0.0009 |
| 44 |  |  | 0.0007 | 0.0007 | 0.0015 | 0.0015 |
| 47 |  |  | 0.0101 | 0.0101 | 0.0202 | 0.0202 |
| 48 |  |  | 0.00436 | 0.00436 | 0.00436 | 0.00436 |
| 51 |  |  | 0.0018 | 0.0018 | 0.0054 | 0.0054 |

Of the 26 products with a Mn salt listed on their labels, 4 (15.4%) exceeded the Institute of Medicine’s UL for 1- to 3-year-olds (Tables 3 and 5). Notably, 3 of these 26 (11.5%) products are identical to the ones whose labeled content equaled the Institute of Medicine’s ULs; see Table 4. An additional product provides enough Mn to exceed the Institute of Medicine’s UL for 4 to 5-year-olds (Tables 3 and 5). This product (sample 20) contained more than twice its labeled value. None of the products with no Mn salt listed on their labels exceeded the Institute of Medicine’s ULs.

***Daily Exposure Estimates Based on Ordinary Dietary Intakes in Addition to Measured Values in Supplements***

Children’s vitamin and mineral products are intended to be consumed as supplements to ordinary intakes from food and beverages. Unfortunately, NHANES does not include Mn intakes in its What We Eat in America (WWEIA) surveys [33], so current nationwide representative surveys of dietary Mn intakes are not available. Nevertheless, the Institute of Medicine constructed its AIs for Mn based on the median recorded intakes of food from earlier surveys [39]. Thus, we can use the Institute of Medicine’s AIs themselves as estimates of median dietary intakes (Table 3). Table 6 presents daily Mn exposure estimates when the products that we tested are consumed as supplements to food in ordinary diets.

Table 6. Total dietary exposure estimates in mg/day for children at different ages from 6 months to 5 years, consuming median dietary intakes of Mn, supplemented with the products containing measurable Mn content. Products that exceed the Institute of Medicine’s tolerable Upper intake Levels (ULs) for corresponding age classes when consumed as supplements in addition to ordinary diets according to measured Mn content are highlighted in **bold**. Samples with the measured Mn content below the detection limit not shown.

| **Sample** | **Total Dietary Intakes of Manganese (Mn) Based on Ordinary Diets and Measured Mn Content** | | | | | |
| --- | --- | --- | --- | --- | --- | --- |
|  | **Daily Intake** | **Daily Intake** | **Daily Intake** | **Daily Intake** | **Daily Intake** | **Daily Intake** |
|  | **6 Months** | **1 Year** | **2 Years** | **3 Years** | **4 Years** | **5 Years** |
|  | **(mg/day)** | **(mg/day)** | **(mg/day)** | **(mg/day)** | **(mg/day)** | **(mg/day)** |
| **1** | 0.6 | 1.2 | **4.02** | **4.02** | **4.32** | **4.32** |
| 2 | 0.6 | 1.2 | 1.2 | 1.2 | 2.14 | 2.14 |
| 3 | 0.6 | 1.64 | 1.64 | 1.64 | 2.38 | 2.38 |
| **4** | 0.6 | 1.2 | **3.45** | **3.45** | **3.75** | **3.75** |
| **5** | 0.6 | 1.2 | **2.68** | **2.68** | **4.45** | **4.45** |
| **6** | 0.6 | 1.2 | 1.2 | 1.2 | **3.75** | **3.75** |
| **7** | 0.6 | 1.2 | **2.36** | **2.36** | **3.82** | **3.82** |
| 8 | 0.6 | 1.2 | 1.2 | 1.2 | 2.42 | 2.42 |
| 9 | 0.6 | 1.2 | 1.2 | 1.78 | 2.08 | 2.08 |
| 10 | 0.6 | 1.87 | 1.87 | 1.87 | 2.17 | 2.17 |
| 11 | 0.6 | 1.2 | 1.2 | 1.2 | 1.84 | 1.84 |
| 12 | 0.6 | 1.2 | 1.2 | 1.2 | 2.63 | 2.63 |
| 13 | 0.6 | 1.2 | 1.2 | 1.2 | 2.78 | 2.78 |
| 14 | 0.6 | 1.2 | 1.2 | 1.2 | 1.66 | 1.66 |
| 15 | 0.6 | 1.2 | 1.22 | 1.22 | 1.53 | 1.53 |
| 16 | 0.6 | 1.2 | 1.2 | 1.22 | 1.53 | 1.53 |
| **17** | **1.53** | **3.05** | **3.05** | **3.05** | **4.28** | **4.28** |
| 18 | 0.6 | 1.32 | 1.32 | 1.32 | 1.62 | 1.62 |
| 19 | 0.6 | 1.2 | 1.81 | 1.81 | 2.11 | 2.11 |
| **20** | 0.6 | 1.2 | 1.2 | 1.2 | **4.60** | **4.60** |
| 21 | 0.6 | 1.2 | 1.2 | 1.2 | 2.45 | 2.45 |
| 22 | 0.6 | 1.2 | 1.40 | 1.40 | 1.70 | 1.70 |
| **23** | 0.6 | 1.2 | **3.47** | **3.47** | **3.77** | **3.77** |
| **24** | 0.6 | 1.2 | 1.2 | 1.2 | **3.42** | **3.42** |
| 25 | 0.6 | 1.2 | 1.82 | 1.82 | 2.74 | 2.74 |
| 26 | 0.6 | 1.2 | 1.2 | 1.2 | 1.5 | 1.98 |
| 27 | 0.6 | 1.2 | 1.22 | 1.22 | 1.54 | 1.54 |
| 30 | 0.6 | 1.20 | 1.20 | 1.20 | 1.50 | 1.50 |
| 31 | 0.6 | 1.2 | 1.20 | 1.20 | 1.50 | 1.50 |
| 33 | 0.6 | 1.2 | 1.52 | 1.52 | 2.15 | 2.15 |
| 35 | 0.6 | 1.2 | 1.21 | 1.21 | 1.52 | 1.52 |
| 37 | 0.6 | 1.2 | 1.20 | 1.20 | 1.50 | 1.50 |
| 38 | 0.6 | 1.2 | 1.20 | 1.20 | 1.50 | 1.50 |
| 41 | 0.6 | 1.2 | 1.2 | 1.2 | 1.53 | 1.53 |
| 43 | 0.6 | 1.2 | 1.20 | 1.20 | 1.50 | 1.50 |
| 44 | 0.6 | 1.2 | 1.20 | 1.20 | 1.50 | 1.50 |
| 47 | 0.6 | 1.2 | 1.21 | 1.21 | 1.52 | 1.52 |
| 48 | 0.6 | 1.2 | 1.20 | 1.20 | 1.50 | 1.50 |
| 51 | 0.6 | 1.2 | 1.20 | 1.20 | 1.51 | 1.51 |

Of the 26 products with a Mn salt listed on their labels, 9 (34.6%) contained enough Mn to exceed the Institute of Medicine’s ULs when consumed as supplements to ordinary diets. None of the products with no Mn salt listed on their labels would lead to exceedances of the Institute of Medicine’s ULs when consumed as supplements to ordinary diets.

We found no difference in cost per day between samples that led to exceedances of the Institute of Medicine’s ULs when consumed as supplements and those that did not (W(52) = 179, *p*=.735).

**Conclusions**

This survey of children’s supplements found that some products contained substances that interfered with the measurement of Mn by spectrophotometry. It is possible that the substances responsible for this interference may affect the bioavailability of Mn and potentially other minerals contained within the supplements.

Most products contain more Mn than reported on their labels, with an average percent difference of 42.5% (Table 2). We found that 50.0% of products that did not list Mn on their labels contained measurable concentrations of this element (Table 2).

Of the products that we tested that did not list Mn on their labels, none would lead to exceedances of the Institute of Medicine’s ULs if consumed in the amounts indicated on the label. In contrast, of the products that we tested with a Mn salt listed on their labels, 15.4% would exceed the Institute of Medicine’s ULs based on their labeled Mn content if consumed with food (Table 4). Of these same products, 19.2% would exceed the ULs based on their measured Mn content, even without additional dietary Mn intake (Table 5). When consumed as supplements to a normal diet, as many as 35% of the products that we tested with Mn reported on their labels would lead to exceedances of ULs (Table 6). The cost of the supplements was not correlated with the Mn content, the percent difference between labeled and measured Mn content, or the likelihood of exceedances of the Institute of Medicine’s ULs.

It must be noted that the Institute of Medicine’s ULs used for comparison were derived from the maximum intakes of just 2 adult individuals in a study that measured no health outcomes [53], so it is quite possible that the ULs used in the comparisons above are not sufficiently protective for children.

While high dietary intakes of Mn have not been correlated with high blood or serum concentrations of Mn in adults [55], supplemental Mn has been shown to increase blood or serum Mn levels [56-58], and high blood and serum Mn levels have been associated with neurodevelopmental and other adverse health outcomes for children [2,20,21]. The Institute of Medicine’s AIs for Mn actually represent average observed intakes since true adequate intake levels have never been determined. Thus, it is quite uncertain whether Mn supplements in children are ever beneficial.

To avoid excess exposure to Mn, parents may be advised to choose multivitamin supplements that are not labeled to contain any Mn, if their pediatricians recommend using multivitamin supplements at all. Manufacturers may be advised to remove Mn from their multivitamin supplements in order to avoid the risk of harm through exceedances of tolerable levels of Mn, or at least reduce the Mn content of their supplements so that the ULs will not be exceeded when the products are consumed alongside ordinary diets.

**Acknowledgements**

We acknowledge Norwich University and the University of Vermont for providing institutional support for this project.

**Author Contributions**

***Conceptualization*:** Seth H. Frisbie

***Data curation*:** Seth H. Frisbie

***Formal analysis*:** Erika J. Mitchell

***Investigation*:** Seth H. Frisbie, Amy Hoeltge, Lindsey A. Pett, Molly G. Hoeltge

***Methodology*:** Seth H. Frisbie, Erika J. Mitchell, Amy Hoeltge, Lindsey A. Pett

***Validation*:** Seth H. Frisbie

***Writing – original draft:*** Erika J. Mitchell, Seth H. Frisbie, Amy Hoeltge, Lindsey A. Pett

***Writing – review & editing*:** Erika J. Mitchell, Seth H. Frisbie, Amy Hoeltge, Lindsey A. Pett

**References**

1. Stierman B, Mishra S, Gahche JJ, Potischman N, Hales CM. Dietary supplement use in children and adolescents aged ≤19 years — United States, 2017–2018. MMWR Morb Mortal Wkly Rep. 2020;69(43):1557-1562. <https://doi.org/10.15585/mmwr.mm6943a1>. PMID: 33119556
2. Liu W, Zin Y, Li Q, Shang Y, Ping Z, Min J et al. Biomarkers of environmental manganese exposure and associations with childhood neurodevelopment: a systematic review and meta-analysis. Environ Health. 2020;19(1):104. <https://doi.org/10.1186/212940-020-00659-x>. PMID: 33008482
3. Lucchini RG, Martin CJ, Doney BC. From Manganism to manganese-induced Parkinsonism: a conceptual model based on the evolution of exposure. Neuromol Med. 2009;11:311-321. <https://doi.org/10.1007/s12012-009-8108-8>. PMID: 20012385
4. Wasserman GA, Liu X, Parvez F, Ahsan H, Factor-Litvak P, Kline, et al. Water manganese exposure and children’s intellectual function in Araihazar, Bangladesh. Environ Health Perspect. 2006; 114:124–129. [https://doi.org/10.1289/ehp.8030](https://doi.org/10.1289/ehp.8030%20) PMID: 16393669
5. Shui W, Niu Y, Zhang C, Pan Q. Investigating the correlation between blood manganese concentrations and anemia in U.S. adults: a nationally representative study. Hematology. 2025;30(1):2460895. <https://doi.org/10.1080/16078454.2025.2460895>. PMID: 39977843
6. Dai G, Sun H, Lan Y, Jiang J, Fang B. The association of manganese levels with red cell distribution width: A population-based study. PLoS ONE. 2024;19(8):e0292569. <https://doi.org/10.1371/journal.pone.0292569>. PMID: 39146304
7. Costa AM, Sias RJ, Fuchs SC. Effect of whole blood dietary mineral concentrations on erythrocytes: selenium, manganese, and chromium: NHANES Data. Nutrients. 2024;16(21):3653. <https://doi.org/10.3390/nu16213653>. PMID: 39519486
8. Ling R, Ai Y, Chen C, Zhang J, Zou Z, Cheng S et al. Risk of environmental chemicals on bone fractures is independent of low bone mass in US Adults: Insights from 2017 to 2018 NHANES. Metabolites. 2023;13(3):346. <https://doi.org/10.3390/metabo13030346>. PMID: 36984786
9. Wei MH, Cui Y, Zhou HL, Song WJ, Di DS, Zhang RY, et al. Associations of multiple metals with bone mineral density: a population-based study in US adults. Chemosphere. 2021;282:131150. <https://doi.org/10.1016/j.chemosphere.2021.131150>. PMID: 34470175
10. Pu B. Association between blood manganese and cardiovascular diseases among U.S. adult population. Sci Rep. 2024;14(1):31260. <https://doi.org/10.1038/s41598-024-82673-4>. PMID: 39733137
11. Zhang J, Wang Y, Jin S, Song N, Zhao S, Fang Y et al. Non-linear associations between serum manganese with chronic kidney disease: results from two nationwide studies in the United States and China. Ren Fail. 2025;47(1):2512049. <https://doi.org/10.1080/0886022X.2025.2512049>. PMID: 40467333
12. Shi X, Wang X, Zhang J, Dang Y, Ouyang C, Pan J et al. Associations of mixed metal exposure with chronic kidney disease from NHANES 2011–2018. Sci Rep. 2024;14(1):13062. <https://doi.org/10.1038/s41598-024-63858-3>. PMID: 38844557
13. Xue Q, Chen H. Association of blood manganese levels with non­alcoholic fatty liver disease in NHANES 2017- 2020: a retrospective cross-sectional study. Metabol Open. 2025;26:100358. <https://doi.org/10.1016/j.metop.2025.100358>. PMID: 40224537
14. Han K, Shen J, Tan K, Liu J, Sun W, Gao Y. The association between blood manganese and liver stiffness in participants with chronic obstructive pulmonary disease: a cross‑sectional study from NHANES 2017–2018. Eur J Med Res. 2023;28(1):14. <https://doi.org/10.1186/s40001-022-00977-5>. PMID: 36611212
15. He Z, Zhao Y, Tang H. Serum manganese and its association with non-alcoholic fatty liver disease: findings from NHANES. Front Nutr. 2025;12:1527207. <https://doi.org/10.3389/fnut.2025.1527207>. PMID: 40225343
16. Alandanoosi A, George F, Liuzzi J. Investigating the relationship among zinc status, Ыооd manganese levels, and enzymatic markers of tissue damage: an epidemiological study using NHANES 2013-2016 data. Toxicol Lett. 2025;403:76-83. <https://doi.org/10.1016/j.toxlet.2024.11.011>. PMID: 39631448
17. Guo W, Weng T, Song Y. Impact of blood lead and manganese levels on metabolic dysfunction-associated steatotic liver disease prevalence: insights from NHANES (2017–2020). BMC Gastroenterol 2025;25(1):160. <https://doi.org/10.1186/s12876-025-03731-3>. PMID: 40069625
18. Liu X, Shen H, Chen M, Shao J. Clinical relevance of environmental manganese exposure with liver stiffness and steatosis detected by transient elastography in adults. Environ Sci Pollut Res Int. 2022;29(11):16624-16632. <https://doi.org/10.1007/s11356-021-17012-5>. PMID: 34651275.
19. Huang Q, Wan J, Nan W, Li S, He B, Peng Z. Association between manganese exposure in heavy metals mixtures and the prevalence of sarcopenia in US adults from NHANES 2011-2018. J Hazard Mater. 2024;464:133005. <https://doi.org/10.1016/j.jhazmat.2023.133005>. PMID: 37988867
20. Fang P, Wang Y, Chen N, Gao K, Gong W, Yang L et al. Association of Ыооd trace metals with anemia in children aged 6-17 years old. J Trace Elem Med Biol. 2025;Jun:89:127655. <https://doi.org/10.1016/j.jtemb.2025.127655>. PMID: 40245650
21. Liu J, Tang Y, Chen Y, Zhang X, Xia Y, Geng B. Association between blood manganese and bone mineral density in US adolescents. Environ Sci Pollut Res Int. 2023;30(11):29743-29754. <https://doi.org/10.1007/s11356-022-24314-9>. PMID: 36422781
22. Polites, MD, Freedman JC, Haynes EN, Sanders AP. Association of manganese biomarker concentrations with blood pressure and kidney parameters among healthy adolescents: NHANES 2013–2018. Children. 2021;8(10):846. <https://doi.org/10.3390/children8100846>. PMID: 34682111
23. You B, Chen Z. Association of blood manganese and selenium levels with hepatic steatosis among adolescents: a nationwide cross-sectional analysis. Front Pediatr. 2025;13:1522219. <https://doi.org/10.3389/fped.2025.1522219>. PMID: 40007873
24. Madrigal JM, Persky V, Pappalardo A, Argos M. Association of heavy metals with measures of pulmonary function in children and youth: Results from the National Health and Nutrition Examination Survey (NHANES). Environ Int. 2018;121(Pt 1):871-878. <https://doi.org/10.1016/j.envint.2018.09.045>. PMID: 30343186
25. Akinkugbe, AA, Midya V, Duffy J, Landero J, Wright RO, Wright RJ. Metal Mixtures and oral health among children and adolescents in the National Health and Nutrition Examination Survey (NHANES), 2017-2020. Int J Hyg Environ Health. 2024;257:114335. <https://doi.org/10.1016/j.ijheh.2024.114335>. PMID: 38330728
26. Wang X, Xiao P, Wang R, Luo C, Zhang Z, Yu S, et al. Relationships between urinary metals concentrations and cognitive performance among U.S. older people in NHANES 2011–2014. Front Public Health. 2022;10:985127. <https://doi.org/10.3389/fpubh.2022.985127>. PMID: 36148349
27. Yang S, Liang Z, Qiu Y, Li X, Tian Y, Liu Y. Association between heavy metals and risk of cardiovascular diseases in US adults with prediabetes from NHANES 2011–2018. BMC Public Health. 2025;25(1):391. <https://doi.org/10.1186/s12889-025-21552-7>. PMID: 39885432
28. Chen X, Gu Z, Qi Y, Hao, X, Xie L. Manganese exposure and its U-shaped relationship with diabetic retinopathy: analysis of NHANES 2011–2020. Front Nutr. 2025;12:1619751. <https://doi.org/10.3389/fnut.2025.1619751>. PMID: 40612319
29. Ou J, Sun Y, Tong J, Tang W, Ma G. The relationship between serum manganese concentration with all‑cause and cause‑specific mortality: a retrospective and population‑based cross‑sectional study. BMC Cardiovasc Disord. 2024;24(1):229. <https://doi.org/10.1186/s12872-024-03872-5>. PMID: 38678176
30. Xu B, Chen ZX, Zhou WJ, Su J, Zhou Q. Associations between blood manganese levels and sarcopenia in adults: insights from the National Health and Nutrition Examination Survey. Front Public Health. 2024;12:1351479. <https://doi.org/10.3389/fpubh>.2024.1351479. PMID: 38803810
31. Tao C, Huang Y, Huang X, Li Z, Fan Y, Zhang Y, et al. Association between blood manganese levels and visceral adipose tissue in the United States: A population-based study. Nutrients. 2022;14(22):4770. <https://doi.org/10.3390/nu14224770>. PMID: 36432456
32. Jiang C, Yi J, Lai J, Wen L, Zhong X, Tan R et al. A U-shaped relationship between blood manganese levels and anemia in patients with CKD: A cross-sectional analysis from National Health and Nutrition Examination Survey 2015 to 2018. Kidney Med. 2025;7(8):101050. <https://doi.org/10.1016/j.xkme.2025.101050>. PMID: 40740730
33. What We Eat in America (WWEI). WWEIA/NHANES and FNDDS - List of Nutrients/Food Components (Unit). Beltsville MD: US Department of Agriculture. Food Surveys Research Group. 2024. Available from: <https://www.ars.usda.gov/northeast-area/beltsville-md-bhnrc/beltsville-human-nutrition-research-center/food-surveys-research-group/docs/wweianhanes-and-fndds-list-of-nutrientsfood-components-unit/>. Cited 1 Oct 2025.
34. National Diet and Nutrition Survey (NDNS). National Diet and Nutrition Survey (NDNS) results on the diet, nutrient intake and nutritional status of adults and children in the UK for 2019 to 2023. London: National Diet and Nutrition Survey. Available from: <https://www.gov.uk/government/statistics/national-diet-and-nutrition-survey-2019-to-2023>. Cited 1 Oct 2025.
35. Huang K, Fang H, Yu D, Guo Q, Xu X, Ju L, et al. Usual intake of micronutrients and prevalence of inadequate intake among Chinese adults: Data from CNHS 2015–2017. Nutrients 2022;14:4714. PMID: 36432400
36. Zipkin F. Assessment of Manganese Dietary lntake for а Rural Pediatric Population. PhD. Dissertation submitted to University of Cincinnati. Available from: <https://etd.ohiolink.edu/apexprod/rws_etd/send_file/send?accession=ucin1396523208&disposition=inline>. Cited 27 July 2023.
37. Sorokina T, Sobolev N, Belova N, Aksenov A, Kotsur D, Trofimova A, et al. Diet and blood concentrations of essential and non-essential elements among rural residents in Arctic Russia. Nutrients. 2022;14(23):5005. <https://doi.org/10.3390/nu14235005>. PMID: 36501035
38. National Institutes of Health (NIH). Manganese: Fact Sheet for Health Professionals. 2021. Washington DC: National Institutes of Health. Available from: <https://ods.od.nih.gov/factsheets/Manganese-HealthProfessional/>. Cited 1 Oct 2025.
39. Institute of Medicine (IOM). Dietary Reference Intakes for vitamin A, vitamin K, arsenic, boron, chromium, copper, iodine, iron, manganese, molybdenum, nickel, silicon, vanadium, and zinc. Washington, DC: United States Institute of Medicine. 2001. Available from: <https://www.ncbi.nlm.nih.gov/books/NBK222310/pdf/Bookshelf_NBK222310.pdf>. Cited 21 Oct 2025. [https://doi.org/10.1016/S0002-8223(01)00078-5.11269606](https://www.ncbi.nlm.nih.gov/books/NBK545442/table/appJ_tab3/?report=objectonly)
40. Hach Company. 1-(2-Pyridylazo)-2-naphthol PAN method. 2014. Available from: <https://www.hach.com/asset-get.download.jsa?id=7639983728>. Cited 24 June 2017.
41. Skoog DA, Holler FJ, Crouch SR. Principles of instrumental analysis. 7th ed. Boston, MA: Cengage Learning; 2018. pp. 231-246.
42. American Public Health Association, American Water Works Association, and Water Environment Federation. Rice EW, Baird RB, Eaton AD, Clesceri LS, editors. Standard methods for the examination of water and wastewater. 22nd ed. Washington, DC: American Public Health Association; 2012. pp. 1–6 to 1–36.
43. Sensoy I. A review on the food digestion in the digestive tract and the used in vitro models. Curr Res Food Sci. 2021;4:308-319. <https://doi.org/10.1016/j.crfs.2021.04.004>. PMID: 34027433
44. International Union of Pure and Applied Chemistry (IUPAC). McNaught AD, Wilkinson A, editors. Compendium of chemical terminology. 2nd ed. Oxford, United Kingdom: Blackwell Scientific Publications; 1997. Nic M, Jirat J, Kosata B, Jenkins A, editors. XML on-line corrected version. 2014. Available from: [http://goldbook.iupac.org/html/L/L03540.html. Cited 27 June 2017](http://goldbook.iupac.org/html/L/L03540.html.%20Cited%2027%20June%202017).
45. Gibbons RD, Coleman DE. Statistical methods for detection and quantification of environmental contamination. New York, NY: John Wiley & Sons; 2001. pp. 48-89.
46. Frisbie SH, Mitchell EJ, Abualrub MS, Abosalem Y. Calculating the lowest reportable concentrations of toxic chemicals in the environment. Int J Appl Mat Theor Phys. 2015;1(1):9-13. <https://doi.org/10.11648/j.ijamtp.20150101.12>.
47. National Institute of Standards and Technology (NIST). Multielement Tablets Standard Reference Material® (SRM 3294). 2021. Available from: <https://tsapps.nist.gov/srmext/certificates/3294.pdf>. Cited 13 December 2021.
48. Andrews KW, Palachuvattil J, Gusev PA, Dang PT, Savarala S, Han F. USDA Dietary Supplement lngredient Database. Release 4.0 (DSID-4). Children’s Multivitamin/mineral (MVM). Dietary Supplement Study. Research Summary. Beltsville MD: US Department of Agriculture. 2017. Available from: <https://dsid-dev.app.cloud.gov/studies/multivitamin-minerals/children-national-study>. Cited 29 Sept 2025.
49. United States Congress. Dietary Supplement Health and Education Act of 1994. Public Law 103-417. 1994. Available from: <https://ods.od.nih.gov/About/DSHEA_Wording.aspx>. Cited 5 Oct 2025.
50. United States Food and Drug Administration (U.S. FDA). Select Committee on Generally Recognized As Safe Substances (SCOGS). 2025. Available from: <https://hfpappexternal.fda.gov/scripts/fdcc/index.cfm?set=SCOGS>. Cited 19 Oct 2025.
51. National Institutes of Health (NIH). Dietary Reference Intakes (DRIs): Recommended Dietary Allowances and Adequate Intakes, Elements. Food and Nutrition Board, National Academies. 2019. Available from: <https://www.ncbi.nlm.nih.gov/books/NBK545442/table/appJ_tab3/?report=objectonly>. Cited 5 October 2025.
52. National Institutes of Health (NIH). Dietary Reference Intakes (DRIs): Tolerable Upper Intake Levels, Elements. Food and Nutrition Board, National Academies. 2019. Available from: <https://www.ncbi.nlm.nih.gov/books/NBK545442/table/appJ_tab9/?report=objectonly>. Cited 5 October 2025.
53. Gibson RS, Scythes CA. Trace element intakes of women. Br J Nutr. 1982;48:241-248. <https://doi.org/10.1079/bjn19820110>. PMID: 6288068
54. Aschner JL, Aschner M. Nutritional aspects of manganese homeostasis. Mol Aspects Med. 2005;26(4-5):353-362. <https://10.1016/j.mam.2005.07.003>. PMID: 16099026
55. Hope S-J, Daniel K, Gleason KL, Comber S, Nelson M, Powell JJ. Influence of tea drinking on manganese intake, manganese status and leucocyte expression of MnSOD and cytosolic aminopeptidase P. Eur J Clin Nutr. 2006;60(1):1-8. <https://doi.org/10.1038/sj.ejcn.1602260>. PMID: 16118651
56. Greger JL, Davis CD, Suttie JW, Lyle BJ. Intake, serum concentrations, and urinary excretion of manganese by adult males. Am J Clin Nutr. 1990;51(3):457-461. <https://doi.org/10.1093/ajcn/51.3.457>.
57. Davis CD, Greger JL. Longitudinal changes of manganese­ dependent superoxide dismutase and other indexes of manganese and iron status in women. Am J Clin Nutr. 1992;55(3):747-52. <https://doi.org/10.1093/ajcn/55.3.747>. PMID: 1550052
58. Medithi S, Dayal Kasa Y, Radhakrishna Kankipati V, Kodali V, Jee B, Jonnalagadda PR. Impact of micronutrient supplementation on pesticide residual, acetylcholinesterase activity, and oxidative stress among farm children exposed to pesticides. Front Public Health. 2022;10:872125. <https://doi.org/10.3389/fpubh.2022.872125>. PMID: 35774575
59. Kousa A, Loukola-Ruskeeniemi, Hatakka T, Kantola M. High manganese and nickel concentrations in human hair and well water and low calcium concentration in blood serum in a pristine area with sulphide-rich bedrock. Environ Geochem Health. 2022;44:3799-3819. <https://doi.org/10.1007/s10653-021-01131-6>. PMID: 34698984
